# Noninvasive physical plasma in vulvar lichen sclerosus: a prospective pilot study

**DOI:** 10.1097/JW9.0000000000000277

**Published:** 2026-06-26

**Authors:** Carolin Schröder, Karla Feodorovici, Laura Tascón Padrón, Lucia A. Otten, Matthias Bernhard Stope, Alexander Mustea

**Affiliations:** a Department of Gynaecology and Gynaecological Oncology, University Hospital Bonn, Bonn, Germany

**Keywords:** chronic inflammatory skin disease, lichen sclerosus, noninvasive physical plasma, vulvar dermatoses

## Abstract

**Background:**

Lichen sclerosus (LS) is a chronic inflammatory dermatosis predominantly affecting the anogenital region and is associated with significant impairment of quality of life. High-potency topical corticosteroids are the standard of care; however, some patients show inadequate response or experience relapse. Noninvasive physical plasma (NIPP) is an emerging therapeutic modality with antimicrobial and anti-inflammatory properties, but its role in LS remains unclear.

**Objective:**

To evaluate the safety, tolerability, and preliminary efficacy of NIPP as an adjunctive treatment in patients with vulvar LS refractory to topical corticosteroids.

**Methods:**

This prospective single-arm pilot study included 5 women with histologically confirmed LS and/or chronic vulvar inflammation with persistent symptoms despite topical corticosteroid therapy. Patients received at least 3 NIPP sessions (plasma care, terraplasma medical GmbH), each applied for 1 minute over a 4 × 4 cm area at intervals of 4 to 6 weeks, while continuing standard topical treatment. Clinical assessment, photographic documentation, and patient-reported outcomes using a study-specific questionnaire were collected at baseline, at each session, and at follow-up 4 to 6 weeks after the final treatment.

**Results:**

The median age was 46 years, and the median disease duration was 3 years. No consistent improvement in symptoms or clinical findings was observed following NIPP treatment. Three patients indicated willingness to undergo NIPP again, and 1 patient reported improvement in sexual well-being. No severe adverse events occurred. Treatment was generally well tolerated; however, 1 patient discontinued participation due to treatment-related discomfort.

**Limitations:**

The main limitations are small sample size, lack of a control group, and heterogeneity in disease duration and prior/current treatments.

**Conclusion:**

In this pilot study, adjunctive NIPP did not demonstrate consistent clinical benefit in vulvar LS but appeared safe and well-tolerated. Larger controlled studies are required to further assess its therapeutic potential.

**What is known about this subject in regard to women and their families?**

- Vulvar lichen sclerosus is a chronic inflammatory condition predominantly affecting women and is associated with substantial physical discomfort, sexual dysfunction, and reduced quality of life.
- High-potency topical corticosteroids are the standard treatment; however, a subset of women remains symptomatic despite long-term therapy, highlighting the need for additional treatment options.

**What is new from this article as messages for women and their families?**

- This is the first prospective pilot study evaluating noninvasive physical plasma as an adjunctive treatment for vulvar lichen sclerosus.
- While noninvasive physical plasma was safe and well-tolerated, no consistent clinical benefit was observed in women with refractory disease, underscoring the need for controlled studies and earlier intervention strategies.

## Introduction

Lichen sclerosus (LS) is a chronic inflammatory skin disorder with a predilection for the anogenital region and a prevalence estimated at approximately 1 in 300 women, particularly affecting postmenopausal individuals. It can also affect children and men.^1–3^ The etiology remains unclear but involves autoimmune mechanisms and genetic predisposition.^4,5^ The influence of the microbiome on LS is also being discussed.^6,7^ The clinical course is typically progressive and may lead to irreversible scarring and anatomical changes. The risk of vulvar squamous cell carcinoma is increased.^8,9^ The condition is associated with significant physical discomfort and psychosocial burden.^1^ The current standard of care consists of lifelong high-potency topical corticosteroids, such as clobetasol propionate, which achieve symptomatic control in the majority of cases and reduce the risk of vulvar squamous cell carcinoma.^9^ If corticosteroids are insufficient, systemic immunosuppressants are used. In small case series, innovative approaches such as platelet-rich plasma, fractional CO2 laser, and adipose-derived stem cell applications have shown promising results.^10–12^ However, some patients remain symptomatic despite adequate therapy, and relapses are frequent.^1^

Noninvasive physical plasma (NIPP) is a new device-based technology. The term NIPP describes the medical application of partially ionized gas (plasma) generated at low temperatures that can be safely applied to living tissue. In the field of plasma medicine, the term cold atmospheric plasma is frequently used and refers to a specific type of NIPP generated under atmospheric pressure and at near-room temperature. Thus, cold atmospheric plasma can be considered a commonly used technical implementation within the broader concept of NIPP. NIPP generates reactive oxygen and nitrogen species, exerting antimicrobial and immunomodulatory effects.^13^ These plasma devices deliver reactive oxygen and nitrogen species to the tissue surface without causing thermal damage, thereby enabling localized biological effects. Previous studies have demonstrated its efficacy and safety in patients with, for example, radiation-induced dermatitis and gingival inflammation, and its cytoreductive effect in prostate cancer cells and in patients with cervical intraepithelial neoplasia.^14–17^ The rationale for applying NIPP in LS is its antimicrobial, anti-inflammatory, and potentially antifibrotic effects. By generating reactive oxygen and nitrogen species, NIPP can modulate immune responses, reduce microbial load, and promote tissue regeneration. Given that LS is driven by chronic inflammation and progressive fibrosis, these mechanisms may be particularly relevant in halting disease progression. To date, no clinical studies have evaluated NIPP in women with LS. This pilot study was designed to explore the feasibility, safety, and potential clinical benefit of NIPP therapy in addition to topical corticosteroids in women with symptomatic vulvar LS.

## Methods and material

We conducted a prospective, single-arm, unicentric pilot study at the Department of Gynaecology and Gynaecological Oncology, University Hospital Bonn. Eligible participants were >18 years with clinically and histologically confirmed vulvar LS or histologically confirmed chronic inflammation, and who reported persistent LS symptoms despite at least 3 months of optimized topical corticosteroid therapy (starting with 12 weeks of daily application, then reducing over 4 weeks to every 2 to 3 days, lifelong, with no end of treatment).

Typical LS symptoms were burning sensation, pruritus, dyspareunia, and vaginal dryness, after excluding other gynaecological causes. Patients with active infection, open wound, history or current malignancy, or who were pregnant, were excluded.

**Fig. 1.**
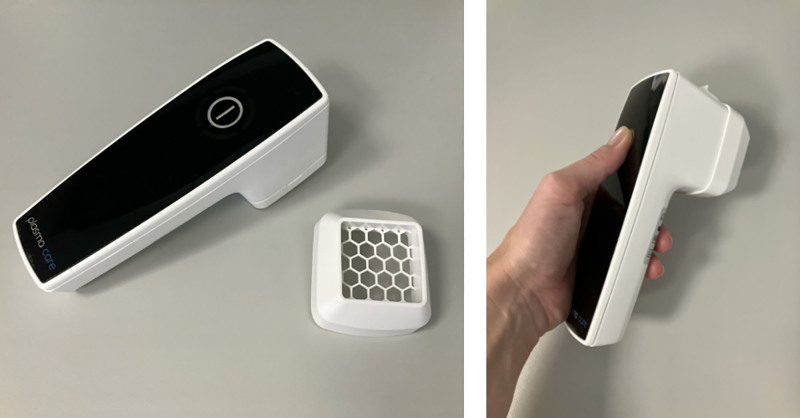
Plasma care device with a 4 × 4 cm spacer (terraplasma medical GmbH).

The intervention consisted of at least 3 NIPP sessions at intervals of 4 to 6 weeks, using the plasma care device with a 4 × 4 cm spacer (terraplasma medical GmbH, Fig. 1). Before treatment, vulvar skin was exposed to 5% acetic acid and visualized under a colposcope to exclude (pre)malignant changes. Since all patients had ubiquitous LS, the entire vulva was treated. Vulvar skin was divided into 4 areas: left, posterior, right, and anterior compartments, which were each treated for 60 seconds using the 4 × 4 cm spacer. The spacer was applied loosely to the skin to ensure a consistent and reproducible distance during each session. The treatment was usually painless and could be performed while the patient was sitting on the gynaecological examination chair.

Since it was designed as a pilot study, the study protocol was developed based on the investigator’s experiences with the NIPP device in previous trials. Participants were instructed to continue their existing topical corticosteroid regimen and other conservative measures (hormonal or nonhormonal topical lubrication, stress reduction in daily life, avoiding tight clothing, etc) throughout the study period and continue this after the study. According to the study protocol, treatments could be extended up to 6 treatment sessions if the patient experienced a benefit or wanted to proceed with the treatment.

Primary endpoints were feasibility and patient-reported outcomes, including changes in symptom severity and quality of life, assessed via a study-specific questionnaire (SSQ) completed by the patient at baseline, at each treatment session, and at the last follow-up 4 to 6 weeks after the last treatment.

The SSQ included items on symptom severity (pruritus, burning sensation, skin atrophy, scarring, and dyspareunia), changes over time, adverse events, impact on daily life, sexual activity, and overall satisfaction with treatment. Responses regarding symptoms were recorded on a Likert scale (none, mild, moderate, and severe). The impact on quality of life and whether the patient would choose the treatment again or recommend it to a friend were also recorded on a Likert scale (strongly agree, somewhat agree, somewhat disagree, and strongly disagree). Regarding sexual activity, patients were asked whether they were sexually active (yes/no) and whether the treatment had a positive impact on their sexual life (yes/no).

Secondary endpoints included clinical evaluation of vulvar skin by an experienced gynecologist and photographic documentation. There was no blinded rating of the photographic documentation. Adverse events were monitored at each visit.

The statistical analysis was performed using the statistical program SPSS 29.0.2.0. 0. 2. 0 (IBM Corp. Released 2023 IBM SPSS Statistics for Windows, Version 29. 0. Armonk, NY: IBM Corp). Descriptive statistics were used to summarize patient characteristics and outcomes, given the small sample size. Ethical approval was obtained from the institutional review board, and all participants provided written informed consent before enrollment. The University of Bonn ethics committee approved the study in June 2024 (reference number 2024-222- BO). The study was registered in the German Clinical Trials Register (DRKS) under the number DRKS 00038762. Initially, this pilot study was designed to include *n* = 20 patients. However, the study was terminated early after treatment of *n* = 5 patients due to lack of subjective and objective efficacy.

## Results

Between September 2024 and May 2025, *n* = 5 female patients were treated at the Department of Gynaecology and Gynaecological Oncology, University Hospital Bonn. The median age of patients was 46 years (range 35–63), with a median body mass index of 24.6 kg/m^2^ (range 20.7–27.6). Three patients were premenopausal (patients 2, 3, and 5) and 2 postmenopausal (patients 1 and 4). The median duration since diagnosis was 3 years, and all patients had received long-term topical corticosteroid therapy before inclusion, most commonly clobetasol, sometimes combined with vaginal lubrication or topical estrogen (Table 1). Histological confirmation of LS was available in 3 cases (patients 1, 2, and 3), whereas 2 patients (patients 4 and 5) showed chronic inflammatory changes without the full histological picture of LS. At baseline, the clinical presentation was characterized by advanced disease stages, with 4 patients showing varying degrees of labial fusion (patients 1, 3, 4, and 5) and 1 patient (patient 2) without overt structural alterations.

**Table 1:** Baseline characteristics and reported outcomes of patients

Patient ID	Age (years)	Body mass index (km/m^2^)	Menopausal status	Disease duration	Main symptom	Previous LS therapy	Histologically LS confirmed	Baseline clinical findings	Reported effect of NIPP	Other
1	55	26.5	Postmenopausal	4 years	Pruritus, burning sensation (3 o’clock)	Topical corticosteroid ointment, topical lubrication (2–3×/week)	Yes	Fusion of the labia minora at 12 o’clock	Increased dryness from the first session on, progressive dyspareunia, no symptomatic improvement	Only the patient with 4 treatment sessions
2	36	27.6	Premenopausal	3 years	Pruritus, burning sensation (6 o’clock)	Topical corticosteroid ointment (1×/week)	Yes	Clinically unremarkable	Increasing paresthesia/tingling on treated areas during and after treatment, no symptomatic improvement	A patient with rheumatoid arthritis treated with adalimumab, the quality of life improved
3	35	20.7	Premenopausal	3 years	Pruritus (12 and 6 o’clock)	Topical corticosteroid ointment (2–3×/week), coconut oil	Yes	Fusion of labia minora, small labia absent	Slight reduction of pruritus, new pain	Cigarette consumption of 13 per day, quality of life, and sexual quality improved
4	63	24.6	Postmenopausal	3 years	Dyspareunia, pain when wearing tight clothes	Topical corticosteroid ointment 3×/week, topical estrogen	Chronic inflammation, no full LS	Fusion of labia minora	Increased sensation of burning at labia majora bilaterally	Would not choose treatment again
5	46	21.0	Premenopausal	4 years	Vaginal dryness	Topical corticosteroid ointment 2–3×/week, topical estrogen	Chronic inflammation, no full LS	Fusion of labia minora, sclerosis of the posterior commissure	Progressive dryness	Discontinuation during third session due to discomfort

LS, lichen sclerosus; NIPP, noninvasive physical plasma.

**Fig. 2.**
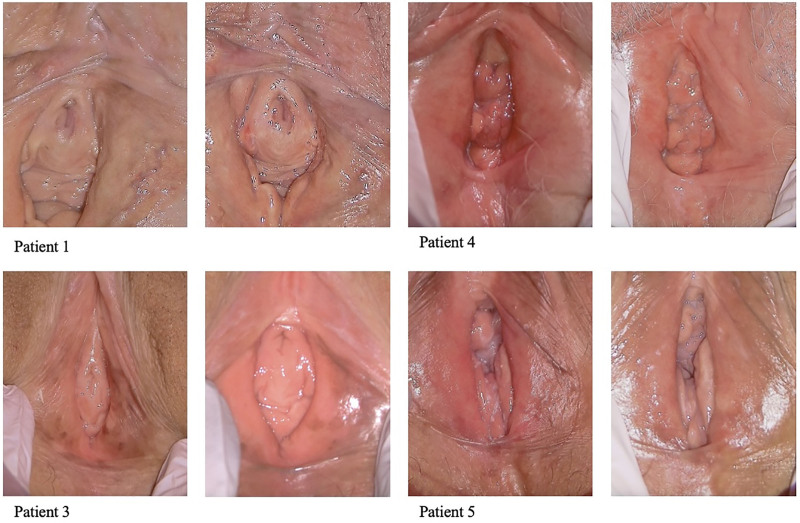
Clinical findings (left: before treatment, right: after treatment).

The predominant symptoms were pruritus and a burning sensation (patients 1, 2, and 3), dyspareunia (patient 4), and marked dryness (patient 5). Patients 2, 3, and 4 received 3 treatment sessions; patient 1 received an additional fourth session. Overall, the intervention was well tolerated. No severe adverse events occurred. One patient reported mild, transient discomfort during treatment without the need for analgesia or discontinuation (patient 2). Patient 5 discontinued treatment during the third session due to discomfort. At each treatment session, a clinical examination using a colposcope was performed. Figure 2 shows that there were no changes in clinical findings between baseline and after the last treatment session.

**Fig. 3.**
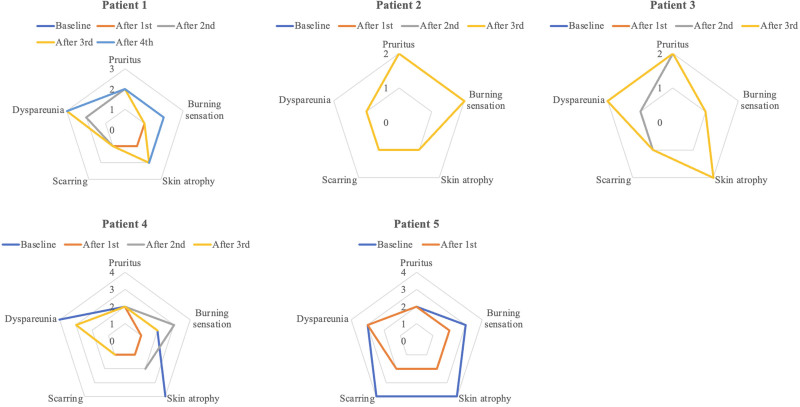
Radar diagram of symptoms (pruritus, burning sensation, skin atrophy, scarring, dyspareunia) with grading of symptoms: 1 = none, 2 = mild, 3 = moderate, 4 = severe.

Patient-reported outcomes obtained through the SSQ revealed a heterogeneous picture. While some patients reported slight improvements in skin atrophy (patients 4, 5) or dyspareunia (patient 4) during the course of treatment, others experienced no meaningful change (patient 2) or even worsening of symptoms (patients 1, 3), such as dyspareunia due to dryness. When patients described a transient benefit, it usually lasted for 5 to 7 days after treatment. Across all patients, there was no consistent trend towards symptom improvement. Overall, the radar charts (Fig. 3) illustrate that symptom trajectories varied individually and did not show a uniform benefit from NIPP therapy. One of the main symptoms patients described during the treatment period was progressive dryness, which explains the persistent or worsening LS symptoms such as pruritus, burning, and dyspareunia.

Three patients (patients 2, 3, and 5) would choose the NIPP treatment again; 2 of them (patients 2 and 3) stated that their quality of life improved slightly after NIPP treatment. All patients were sexually active; only patient 3 noted that the treatment had a positive influence on her sexual life. All patients would recommend the treatment to a friend with similar symptoms.

## Discussion

This pilot study is the first to investigate the use of NIPP in vulvar LS. Although the intervention was safe and feasible, no clinically meaningful benefit was observed in this small cohort of patients with advanced disease refractory to standard topical corticosteroid therapy. Several reasons might explain this.

The study protocol was based on the investigator’s experience, as there was no previous study treating vulvar LS with NIPP. No subjective or objective benefit was observed after 1 or 2 treatment sessions. When patients reported a benefit, it was only temporary, lasting 5 to 7 days. There was therefore no rationale to shorten the treatment intervals when patients described progressive dryness after 2 sessions. Furthermore, the absence of a control group and the small sample size preclude definitive conclusions regarding efficacy. The evaluation of NIPP treatment was based on patient-reported outcomes using the SSQ, which is an important tool for assessing quality of life and impairment of daily activities. Interestingly, some patients reported a temporary improvement in quality of life or sexual well-being despite the absence of objective clinical changes. This discrepancy highlights the role of patient expectations and subjective perception in the evaluation of new therapeutic approaches. Incorporating validated patient-reported outcome measures and qualitative interviews in future trials may help better evaluate these aspects. Hence, we assume that we did not use the right variables to fully evaluate the benefit of NIPP. We did not perform additional histological assessments with NIPP due to the hypothesis-generating study design. Furthermore, due to the short follow-up period, we do not know the influence on vulvar carcinoma risk. As NIPP has been shown to modulate inflammatory and regenerative pathways in patients with cervical intraepithelial neoplasia or in prostate cancer cells, future studies should integrate tissue biopsies and molecular analyses to explore the underlying biological effects in LS. Most patients had long-standing disease with established scarring and/or advanced fibrotic changes, which are unlikely to be reversible through NIPP. It therefore remains purely hypothetical. It is suggested that patients in earlier stages of LS, where inflammatory rather than fibrotic processes predominate, may benefit from NIPP therapy, either as monotherapy or in combination with established treatments such as topical corticosteroids. This is, however, challenging in our daily clinical practice, since LS is an underdiagnosed and undertreated condition, and it still takes many years for women to be diagnosed and treated properly. Even though the median duration of disease was 3 years, 4 of the patients already had irreversible anatomical changes, indicating that the real disease duration must have been much longer. NIPP has shown promising results in other chronic inflammatory and infectious dermatologic conditions by reducing microbial load, enhancing wound healing, and modulating inflammatory mediators.^13^ In LS, which involves a complex interplay of immune dysregulation and fibrosis, these effects might be more relevant in early disease stages before irreversible structural damage occurs. Previous reports of regenerative therapies such as platelet-rich plasma and adipose-derived stem cell applications suggest that targeted interventions can improve symptoms and histologic markers of inflammation in LS.^10–12^ Whether NIPP could provide similar benefits in a different disease phase remains an open question. Moreover, a potential interaction with concomitant corticosteroid use cannot be excluded, as steroids may either mask or attenuate possible treatment effects of NIPP. Dryness was one of the main progressive symptoms under treatment, explaining why NIPP did not have a positive impact on typical LS symptoms such as pruritus, burning, and dyspareunia, even though all patients used additional topical ointments. Further studies should include a standardized treatment plan for the prevention and treatment of vaginal dryness during NIPP treatment. The main limitations of this study include its small sample size, lack of a control group, and heterogeneity in disease duration and prior/current treatments. These limitations underscore the need for larger, randomized controlled trials evaluating NIPP as monotherapy or in combination with standard corticosteroids, particularly in patients with early or moderate disease severity.

## Conclusion

In this pilot study, NIPP therapy was safe but did not yield meaningful clinical or symptomatic improvement in refractory vulvar LS. Further research with adequately powered, controlled designs and optimized treatment protocols is warranted to assess whether NIPP can play a role in the management of LS.

## Conflicts of interest

M.B.S. is the founder and CEO of 5’-TOP-3’ Physical Plasma Medicine. The sponsors had no role in the design, execution, interpretation, or writing of the study. The remaining authors declare that no funds, grants, or other support were received during the preparation of this manuscript.

## Funding

Terraplasma medical GmbH provided the device and disposable materials.

## Study approval

This study was registered in the German Clinical Trials Register (DRKS) under the number DRKS00038762.

## Author contributions

CS: Project administration, data curation, formal analysis, and original draft preparation. KF, LTP, and LAO: Review and editing and visualization (equal). MBS: Funding acquisition, methodology, and review and editing. AM: Project administration, review and editing, and supervision.

## Patient consent

Informed consent to participate was obtained from all of the patients. The patients agreed to the publication of the data, including photos.

## Data availability

Dataset and materials used in this study are available from the corresponding author upon reasonable request in accordance with ICMJE data sharing policies.

## Ethics approval

This study was conducted in compliance with the Declaration of Helsinki. The study was approved by the ethics committee of the University of Bonn with the reference number 2024-222-BO in June 2024.
